# Functional Foods in Preventing Human Blood Platelet Hyperactivity-Mediated Diseases—An Updated Review

**DOI:** 10.3390/nu16213717

**Published:** 2024-10-30

**Authors:** Asim K. Duttaroy

**Affiliations:** Department of Nutrition, Institute of Basic Medical Sciences, Faculty of Medicine, University of Oslo, 0313 Oslo, Norway; a.k.duttaroy@medisin.uio.no

**Keywords:** blood platelet, functional food, phytochemicals, bioactive, cardiovascular diseases, cancer, water-soluble tomato extract, Fruitflow^®^, polyphenols, omega-3 fatty acids, hypertension, kiwifruit extract, platelet aggregation, diabetes

## Abstract

Backgrounds/Objectives: Abnormal platelet functions are associated with human morbidity and mortality. Platelets have emerged as critical regulators of numerous physiological and pathological processes beyond their established roles in hemostasis and thrombosis. Maintaining physiological platelet function is essential to hemostasis and preventing platelet-associated diseases such as cardiovascular disease, cancer metastasis, immune disorders, hypertension, diabetes, sickle cell disease, inflammatory bowel disease, sepsis, rheumatoid arthritis, myeloproliferative disease, and Alzheimer’s disease. Platelets become hyperactive in obesity, diabetes, a sedentary lifestyle, hypertension, pollution, and smokers. Platelets, upon activation, can trawl leukocytes and progenitor cells to the vascular sites. Platelets release various proinflammatory, anti-inflammatory, and angiogenic factors and shed microparticles in the circulation, thus promoting pathological reactions. These platelet-released factors also maintain sustained activation, further impacting these disease processes. Although the mechanisms are unknown, multiple stimuli induce platelet hyperreactivity but involve the early pathways of platelet activation. The exact mechanisms of how hyperactive platelets contribute to these diseases are still unclear, and antiplatelet strategies are inevitable for preventing these diseases. Reducing platelet function during the early stages could significantly impact these diseases. However, while this is potentially a worthwhile intervention, using antiplatelet drugs to limit platelet function in apparently healthy individuals without cardiovascular disease is not recommended due to the increased risk of internal bleeding, resistance, and other side effects. The challenge for therapeutic intervention in these diseases is identifying factors that preferentially block specific targets involved in platelets’ complex contribution to these diseases while leaving their hemostatic function at least partially intact. Since antiplatelet drugs such as aspirin are not recommended as primary preventives, it is essential to use alternative safe platelet inhibitors without side effects. Methods: A systematic search of the PUBMED database from 2000 to 2023 was conducted using the selected keywords: “functional foods”, “polyphenols”, “fatty acids”, “herbs”, fruits and vegetables”, “cardioprotective agents”, “plant”, “platelet aggregation”, “platelet activation”, “clinical and non-clinical trial”, “randomized”, and “controlled”. Results: Potent natural antiplatelet factors have been described, including omega-3 fatty acids, polyphenols, and other phytochemicals. Antiplatelet bioactive compounds in food that can prevent platelet hyperactivity and thus may prevent several platelet-mediated diseases, including cardiovascular disease. Conclusions: This narrative review describes the work during 2000–2023 in developing functional foods from natural sources with antiplatelet effects.

## 1. Introduction

**Figure 1:**
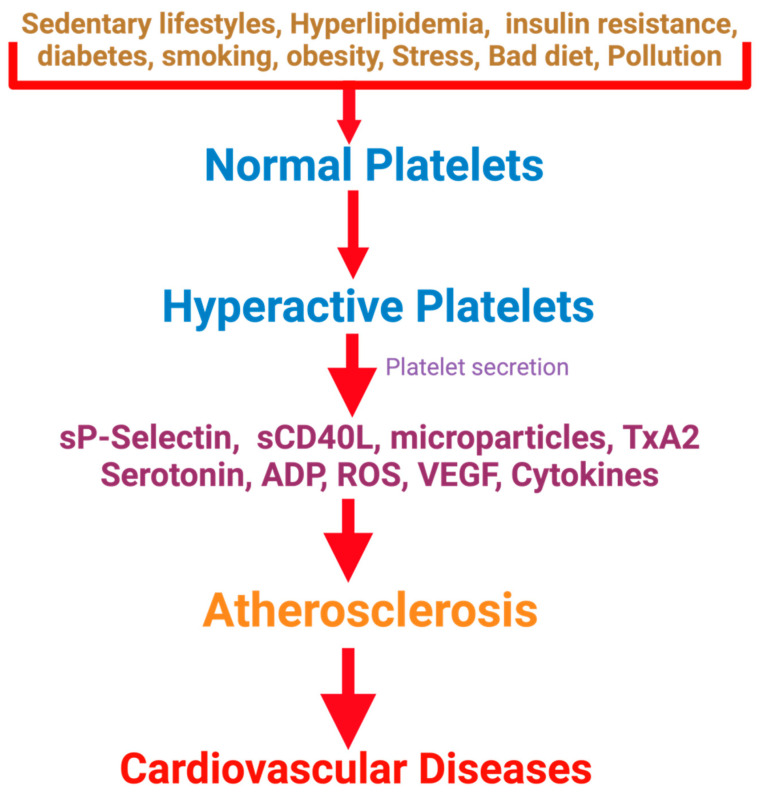
Hyperactivity of platelets and its impact on cardiovascular health.

Maintaining the physiological function of blood platelets is crucial for overall hemostasis and several physiological processes [1,2]. Reduced activation response of blood platelets can lead to excessive bleeding, whereas hyperactivation of platelets contributes to thrombotic complications. Platelet hyperactivity is associated with several processes, such as inflammatory and immune responses, angiogenesis, atherosclerosis, regeneration of liver tissue, and cancer metastasis [3,4,5,6]. Platelet hyperactivity is related to cardiovascular disease (CVD) and many other human diseases, such as cancer, renal diseases, Alzheimer’s disease, and microorganism infections. However, the mechanisms are not well known [4,6,7,8]. The Framingham Heart Study revealed that persistent platelet hyperactivity is also associated with future arterial and venous thrombosis [9]. Individuals with diabetes, a sedentary lifestyle, obesity, and insulin resistance have platelet hyperactivity, which contributes to atherosclerotic plaque development and other platelet-mediated diseases [10,11,12]. Figure 1 depicts the association of platelet hyperactivity with CVD. Platelet hyperactivity is not only associated with CVD and stroke because platelet inhibitors also slow down cancer progression and related morbidities [13]. Platelets play critical roles in inflammation and immune responses [14,15] by supporting the release of IL-6 and NFkB [16], circulating platelet microparticles [17,18], and managing CRP levels [15,19]. A hyperinflammatory status can compromise the body’s response to external stresses and predispose it to diabetes or atherosclerosis.

Platelets play an essential role in CVD, both in the pathogenesis of atherosclerosis and in the development of acute thrombotic events. Although the mechanisms are unknown, platelets become hyperactive in insulin resistance, diabetes mellitus, obesity, smoking, dysbiosis, high-fat diet, pollution, and hyperlipidemia. Hyperactive platelets are involved in developing atherosclerosis through different mechanisms, such as membrane shedding, growth factor secretion, and the expression of several adhesive factors. In addition, hyperactive platelets are involved in the well-known penultimate thrombotic events.

Beyond the hemostatic process, platelets are involved in many processes through complicated interactions with many cells. Aspirin is the most used antiplatelet therapy for the secondary prevention of CVD [20], even though it causes several serious side effects. There are several reasons, including the fact that 25–30% of people are aspirin-resistant and the side effects, making aspirin unsuitable for the primary prevention of CVD [21]. Aspirin prophylaxis is not recommended in individuals without CVD, as per the European Guidelines on CVD prevention [22].

Maintaining regular platelet activity is critical in preventing platelet-associated diseases such as CVD, cancer metastasis, immune disorders, hypertension, diabetes mellitus, sickle cell disease, inflammatory diseases, bowel disease, rheumatoid arthritis, myeloproliferative disease, and Alzheimer’s disease [23,24,25,26,27,28]. Understanding the causes and mechanisms of action of the modifying factors of platelet hyperactivity is also critical in preventing these diseases. 

**Figure 2:**
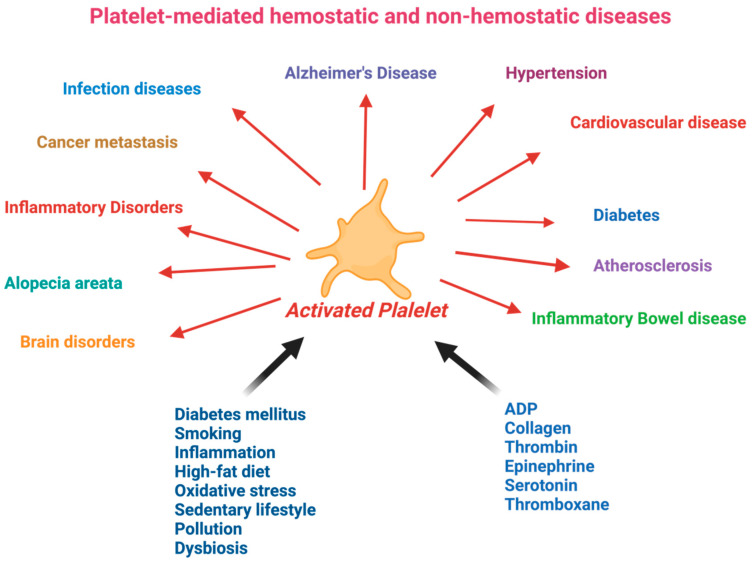
Roles of platelets in several hemostatic and non-hemostatic pathways.

Consequently, there is growing interest in naturally occurring antiplatelet inhibitors that people can regularly consume. Many dietary antiplatelet components have been identified that can reduce platelet hyperactivity without side effects [29,30,31,32,33]. Dietary phytochemicals with antiplatelet activity become operative when continued platelet hyperactivity is experienced in response to inappropriate nutritional and lifestyle factors or exposure to smoke/air pollution [34,35,36], hyperglycemia and hyperlipidemia [37], and specific exercise patterns [38]. Earlier, we reviewed the mechanisms of diverse inhibitory action of various phytochemicals on platelet aggregation [4]. The bioactive compounds affect blood platelet function in multiple ways. Dietary omega-3 LCPUFAs modulate platelet function via TxB3 synthesis, modulation of platelet membranes, and increasing NO synthesis, whereas polyphenols suppress platelet activity via antioxidant activity and/or the GPIV-mediated pathway [4,29,32]. The mechanisms for individual phytochemicals are discussed in a later section. Figure 2 shows that platelet hyperactivity is related to hemostatic and non-hemostatic diseases. The relevance of platelet hyperactivity in the context of many diseases, including the growing epidemic nature of CVD, has strongly suggested the use of more natural approaches in the primary prevention of degenerative diseases. This narrative review provides an integrated summary of recent developments in platelet function in pathophysiology that connect hemostasis and other diseases. This narrative review describes the development of functional foods with antiplatelet factors during 2000–2023. A systematic search of the PUBMED database from 2000 to 2023 was conducted using the selected keywords: “functional foods”, “polyphenols”, “fatty acids”, “herbs”, “fruits and vegetables”, “cardioprotective agents”, “plant”, “platelet aggregation”, “platelet activation”, “clinical and non-clinical trial”, “randomized”, and “controlled”. This review highlights the development of bioactive compounds and their roles in preventing the hyperactivity of blood platelets and associated diseases. It suggests that targeting platelet hyperactivity early with antiplatelet regimes may prevent CVD and other diseases.

Platelets are versatile cells engaged in numerous pathophysiological processes, including inflammation and immunity, angiogenesis, regeneration, and carcinogenesis. Platelets become hyperactive under different conditions, and hyperactive platelets contribute to different diseases, as described in this diagram. Platelets are also crucial in thrombosis and hemostasis via various molecular and cellular events. This figure introduces the emerging role of platelets in the immune system, vascular biology, tumorigenesis, and beyond.

## 2. Platelet Hyperactivity in Many Pathological Conditions

Human blood platelets are involved in hemostasis and thrombosis, including inflammation, infection, immunobiology, cancer metastasis, wound repair, and angiogenesis [39]. Platelets’ vast capacity to regulate various physiological and pathological processes is primarily due to their granular storage and secretion of a wide range of biologically active agents and microparticles [40]. In addition, various platelet subpopulations exhibit biochemical and functional variability impacting these processes [41]. Therefore, multiple functions of platelets are possibly due to the apparent modulation of functions. Thus, platelets are no longer considered only for their roles in the hemostatic system. Platelet functional tests, such as platelet aggregation response and closure time, etc., are used to investigate the possible role of platelet hyperreactivity in the pathogenesis of vascular disorders and their complications.

Since platelet hyperreactivity increases in hyperlipidemia, hyperglycemia, oxidative stress, and cancers [42,43], understanding how platelets become hyperresponsive and contribute to CVD under these conditions is needed to prevent CVD [44]. Platelets maintain endothelial integrity; however, when activated, they disturb endothelium by interacting with their secretions [45,46]. Activated platelets secrete mRNA, nucleotides, enzymes, metal ions, microparticles, mitochondria, ATP, ADP, growth factors, chemokines, cytokines, protease inhibitors, adhesive glycoproteins serotonin, histamine, γ-aminobutyric acid, glutamate, epinephrine, dopamine, histamine, factor V, calcium, and many others [47,48,49]. Platelet microvesicles contain multiple RNAs and proteins involved in these processes [50,51]. In addition, platelets secrete microparticles and microvesicles that are empowered with angiogenic, immunosuppressive, and survival abilities [52,53,54]. The release of these factors by the activated platelets changes the cell membrane architecture and cell shape, including blood vessel walls, affecting the immediate milieu [30]. Platelet activation, aggregation, and the subsequent generation of an occlusive intra-arterial thrombus are essential in developing CVD and platelet-mediated diseases [55,56]. The most harmful consequence of sustained platelet hyperactivity is the widespread deposition of the micro aggregates on the vessel walls. Therefore, lowering baseline platelet activity may be necessary to impair platelet hyperactivity-mediated diseases.

### Pathological Conditions Such as Metabolic Syndrome, Insulin Resistance, Obesity, Hypertension, and Tumors Enhance Platelet Hyperactivity

Platelets are activated by many agonists released in the circulation during pathologic conditions [57]. Platelet hyperactivity is also an underlying risk for diabetes, smoking, obesity, a sedentary lifestyle, pollution, dysbiosis, and other conditions. Inflammation may be exacerbated in patients with hypertension, CVD, and obesity, all associated with baseline platelet hyperreactivity [30,58]. Although the exact mechanism of platelet hyperactivity is not well known in these conditions, it could result from the sensitization of blood platelets to activation/aggregation by agonists [59] and/or due to the redistribution of young, more reactive platelets in the spleen [59] released into the circulation.

Metabolic syndrome (MetS) is a cluster of conditions related to abdominal obesity, insulin resistance, hyperlipidemia, hypertension, and inflammation [60]. MetS is associated with increased platelet reactivity, probably caused by associated hyperglycemia, dyslipidemia, and low-grade systemic inflammation. Platelet hyperactivity in MetS is induced by inflammation, obesity, dyslipidemia, hypertension, oxidative stress, and other metabolic disease [61]. Higher plasma levels of fibrinogen, PAI-1, thrombin, von Willebrand factor, factor VII, and other coagulation factors, as well as significant platelet aggregation, are also observed in MetS [62].

The MetS components increase platelet activation by increasing the calcium concentration and activating arachidonic acid, 20:4n-6 (ARA) signaling pathways. Platelet hyperactivity in dyslipidemia contributes to the thrombotic risk [63]. Platelet activation in dyslipidemia is induced via classic agonist receptor signaling pathways and pattern recognition receptors [63]. Platelets are stimulated by oxidized phospholipids present in low-density lipoproteins (LDLs) via specific pattern recognition receptors [64]. CD36/fatty acid translocase is platelets’ scavenger receptor for oxidized lipids [64].

Oxidative stress may promote platelet hyperactivity by reducing physiologically accessible NO in MetS. Cigarette smoking and sedentary lifestyles also increase oxidative stress and platelet hyperactivity [35]. Platelets oxidize LDL by stimulating the production of reactive oxygen species (ROS) and degrading the LDL receptors by releasing proprotein convertase subtilisin/kexin type 9. Thus, platelet dysfunction and hyperlipidemia stimulate atherogenesis [65]. Moreover, reduced antiplatelet response to aspirin and higher platelet sensitivity toward agonists are observed in MetS. Therefore, hypolipidemic drugs modulate platelet function, whereas antiplatelet drugs normalize lipid metabolism. High levels of plasma leptin in obesity cause increased platelet aggregability [66]. The overall effect of MetS and obesity contributes to platelet activation [67]. Certain adipokines directly influence platelet function and, therefore, have the potential to serve as essential mediators of the cardiovascular consequences of obesity. Consistent with this, obesity is associated with elevations in platelet activation markers such as P-selectin expression and increased platelet microparticle shedding, resulting in increased platelet aggregation. The decreased adipose mass normalizes the markers of enhanced platelet activation. However, a causal role for platelet hyperactivity in obesity-related cardiovascular disorders is yet to be established.

Platelet function is influenced by ROS and nitric oxide (NO) metabolism [68,69]. Platelet aggregation is associated with increased glutathione disulfide levels and oxygen utilization. Changes may influence platelet-dependent thrombus formation in platelet or vascular redox status, antioxidants, ROS, and nitrogen species [70] creation. Hyperglycemia, dyslipidemia, and insulin resistance in diabetic conditions induce platelet hyperactivity, activation of the vascular wall, inflammation, and endothelial dysfunction. Hyperglycemia also causes the overproduction of superoxide and activates protein kinase C and nuclear factor kB (NFkB) [71]. NFkB stimulates the production of several inflammatory cytokines and increases cell adhesion molecules, such as CD40 ligand (CD40L). CD40L induces the production and release of proinflammatory cytokines [72]. Activated platelets express CD40L, indicating a link between hemostasis and inflammation [73].

Diabetic platelets have an increased intrinsic activation profile, whereas these platelets have the reduced influence of endogenous inhibitors and drugs. Thus, platelet hyperactivity in diabetes mellitus results from hypersensitivity to agonists and loss of the antiaggregatory mechanism. Diabetics’ platelet hyperactivity is associated with increased circulating platelet microparticles and hypercoagulability [37]. Even though the usefulness of antiplatelet drugs in type 2 diabetes is not supported, diabetic patients still use low-dose aspirin or other antiplatelet drugs as a potential therapeutic therapy [74,75]. In diabetes mellitus, there is an imbalance between platelet inhibitors such as NO and prostacyclin (PGI_2_) and increased production of platelet-activating and vasoconstricting substances. Decreased endothelium synthesis of PGI_2_ [76] and reduced synthesis of NO was observed in type 2 diabetes mellitus [77]. Platelets express receptors for adhesion and increased synthesis of TxA_2_, thrombin, and deranged calcium metabolism in diabetes. Platelets shed more platelet microparticles in type 2 diabetics and thus may play a role in inflammation and thrombosis. P-selectin-positive platelet microparticles may aid in the recruitment and adhesion of leucocytes, and platelets initiate the atherosclerosis process.

Spontaneous platelet activation is involved in hypertension [78,79,80]. High blood pressure also stimulates platelet activation via increasing the shear force on circulating platelets. Increased blood viscosity in patients with high blood pressure might also contribute to platelet hyperactivity. Under high-pressure flow, enhanced degranulation of platelets occurs [81]. Atherosclerotic lesions induce platelet activation, possibly due to the dysfunctional endothelium and local flow disturbances [82]. Numerous metabolic and physiologic changes occur in platelet reactivity in hypertension. Indeed, many studies have shown that various parameters of platelet activation are normalized with the treatment of hypertension [83]. Platelets from hypertensive patients tend to form aggregates [84], increased secretion of plasma β-thromboglobulin [85], high soluble and membrane expression of P-selectin [86], and increased intracellular levels of calcium [87]. However, the association between these markers and the degree of hypertension or responses to therapy are not well known [88]. Platelet activation and aggregation are also involved in the development of hypertension in different ways. Activated platelets release mediators, such as 5-hydroxytryptamine (5-HT or serotonin), ADP, ATP and lysophosphatidic acid [89]. Several of these agents enhance the intracellular Ca^2+^ concentration in vascular smooth muscle cells, promoting vasoconstriction and increasing the response of catecholamines.

Furthermore, the number of platelet α-adrenergic receptors increases in hypertension [90], which increases catecholamine responses. Catecholamines and angiotensin II increase intracellular Ca^2+^ concentration and promote contraction of vascular smooth muscle cells, platelet activation, and aggregation [90], which may participate in the genesis and maintenance of hypertension. Angiotensin II (Ang II) increases intracellular Ca^2+^ concentration and pH in platelets, which increases platelet sensitivity to aggregating agents [91,92,93]. Hypertensive platelets produce more reactive oxygen species (ROS) [85] that enhance platelet activity by reducing the bioavailability of NO and enhancing intracellular Ca^2+^ concentration. Inflammation is also exacerbated in hypertension, CVD, and obesity, all associated with baseline platelet hyperreactivity [30,58]. 

Endothelial dysfunction is associated with the reduced production of platelet inhibitors and vasodilators, and NO [94]. Therefore, the reduced synthesis of NO increases hypertension and platelet hyperactivity [95]. Similarly, dysfunctional endothelium produces less PGI_2_ and increases platelet activity. Dysfunction of the endothelium in hypertension has a cross-talk with hyperactive platelets [83]. Similarly, increased platelet activation/aggregation in response to endothelin could contribute to thrombosis [96]. Hyperactive platelets are a critical earlier trigger for cancer-associated thrombotic events [97,98]. Interactions between platelets and tumor cells result in hyperactivity or activation of circulating blood platelets.

## 3. Modulation of Platelet Activity by Bioactive Compounds of Dietary Origin

Since the blockade of crucial platelet-activation pathways may prevent platelet hyperreactivity [99], a dietary antiplatelet agent is considered suitable for suppressing platelet hyperactivity in non-CVD people. Antiplatelet drugs would be inappropriate in such a public health setting. Reducing platelet function during the early stages could significantly impact the number of diseases. Natural antiplatelet functional foods have been developed to target platelet hyperactivity early and they have been investigated and may be able reduce several diseases. Nutritional modification of cellular functions by dietary lipids and other nutritive and non-nutritive factors offers an attractive avenue to correct, modify, or prevent many pathophysiological processes, including platelet hyperactivity [100]. Indeed, extensive in vitro and preclinical research demonstrates the efficacy of various food components in reducing platelet hyperactivity. The antiaggregatory effects of different aqueous fruit extracts on human platelets in vitro have been published previously [29]. However, there are only a small number of well-designed studies consistently demonstrating an antiplatelet benefit of only a few select dietary ingredients in healthy human subjects with measurable clinical outcomes. These include fish and fish oil, flavonoids in cocoa, and garlic. Various studies demonstrated that polyphenol supplementation affects platelet aggregation and function in vitro and in vivo, mainly neutralizing free radicals, inhibiting platelet activation via related signal transduction pathways, blocking TxA_2_ receptors, and enhancing NO production. The effect of dietary polyphenols on platelet aggregation in vivo is often conflicting. Only flavanols mirrored in vivo showed efficacy in modulating platelet function in vitro [101]. In addition to fish oils, some plant oils may inhibit blood platelet activation. Since some plant oils contain linolenic acid, 18:3n-3, and phytosterols can prevent platelet hyperactivity [102] but require more human intervention trials for further confirmation.

Therefore, this review’s objectives are two-fold: summarize the available clinical science to substantiate the antiplatelet efficacy of these ingredients and highlight recent and emerging clinical evidence for a new dietary antiplatelet component derived from fruits, vegetables, and fish oils. The antiplatelet benefits of these ingredients on humans, along with clearly defined outcomes related to the primary prevention of vascular events, namely platelet function, are discussed. Therefore, there is an increased interest in identifying bioactive compounds from edible sources with beneficial health effects, efficiency, and fewer adverse effects [4,30,103].

## 4. Omega-3 Fatty Acids

Since the first cross-cultural epidemiological studies in the 1970s, the CVD-preventive role of omega-3 long-chain polyunsaturated fatty acids (LCPUFAs), in particular docosahexaenoic acid, 22:6n-3 (DHA), and eicosapentaenoic acid, 20:5n-3 (EPA), has emerged [104]. Fish oil supplementation reduced platelet aggregation [105]. Essential fatty acids, linoleic acid, 18:2n-6 (LA), and alpha-linolenic acid, 18:3n-3 (ALA), in turn, compete for desaturase and elongase enzymes for their metabolism to LCPUFAs. A higher dietary intake of ALA compared to LA leads to an enhanced synthesis of EPA and DHA metabolites, resulting in a higher production of TxA_3_ than the proaggregatory ARA-derived TxA_2_. Prostaglandin H_2_ is a precursor of other prostaglandins and thromboxane [106]. Antiplatelet mechanisms of action by DHA and EPA are thought to be mediated via inhibition of phospholipase A_2_ activity, which would diminish the liberation of ARA from platelet plasma membrane phospholipids; inhibition of platelet cyclooxygenase 1 (COX-1), which would decrease the conversion of ARA to TxA_2_; competition with ARA for platelet COX-1 and formation of less active TxA_3_, or by alteration of platelet membrane fluidity state; and inhibition of TxA_2_ induced platelet aggregation and decreased receptor affinity for TxA_2_ [107].

Omega-3 LCPUFAs have gained considerable attention for their ability to improve cardiovascular health and prognosis. In contrast, the n-6 fatty acids such as linoleic acid, 18:2n-6, and ARA may increase platelet aggregation. Both EPA and DHA are incorporated into platelet membrane phospholipids, replacing ARA, thus decreasing platelet aggregation via reduced production of ARA-derived platelet-aggregating eicosanoids [29,108]. EPA also competes with ARA for COX, reducing ARA-derived metabolites. Therefore, EPA minimizes the synthesis of the proaggregatory ARA-derived TXA_2_ [109,110]. EPA and DHA also alter membrane fluidity, regulating receptor expression and thrombin generation [111,112,113]. Resolvins produced from both EPA (resolvins E) and DHA (resolvins D) reduce and inhibit thromboxane-induced platelet aggregation [114]. Oxylipins (11-HDHA and 14-HDHA) derived from DHA also inhibited protein kinase A-mediated platelet activation and regulated agonist-induced platelet aggregation [115]. DHA and its 12-LOX-derived oxylipins also modulated collagen-induced platelet aggregation.

Several studies, such as in vitro, clinical trials, observational, epidemiological, and animal studies, investigated the impact of seafood consumption and omega-3 fatty acids on CVD. Human clinical studies showed that omega-3 LCPUFAs impart antiplatelet effects. Fischer et al. (1983) [116] showed that EPA supplementation at 4 gm per day through cod liver oil in eight healthy adult males for 25 days reduced platelet aggregation. In contrast, Gibney and Bolton-Smith (1988) [117] demonstrated that consumption of a low dose of EPA (2.25 gm) plus DHA (1.35 gm) per day did not affect platelet aggregation in response to collagen or low levels of ADP in eight healthy adult males for six weeks. However, there was a significant increase in platelet aggregation when higher levels of ADP were used.

Additionally, platelet TxB2 production was affected [117]. In contrast to other studies, the lack of DHA and EPA effect on platelet function may be due to the low dose of the omega-3 fatty acids used in this study. In a randomized, double-blind study by Li and Steiner (1991), 3, 6, or 9 gm EPA per day supplementation to fifteen healthy adults for three weeks reduced platelet adhesion to fibrinogen within the first week. Under low shear rates conditions, the 3 and 6 gm per day inhibited platelet adhesion by 81.7% and 85.5%, respectively, and the 9 gm dose inhibited rates of platelet adhesion at a similar level to the 3 gm dose. In contrast, platelet adhesion was maximally inhibited at high shear rates by the 3 gm of EPA per day [118].

Pirich and colleagues (1991) also demonstrated that supplementing twenty healthy adult subjects with EPA (216 mg) plus DHA (140 mg) per day for six weeks reduced TxB2 synthesis and significantly increased platelet survival time [119]. Indeed, the levels of DHA and EPA used were remarkably low, considering the positive benefits observed. However, this study’s fish oil supplementation regimen also provided 390 mg of gamma-linolenic acid, 18:3n-6. So, whether the effects observed above were due to the combination of DHA and EPA or the three fatty acids is unclear.

In 2002, Svaneborg and colleagues showed that 10 gm of fish oil per day to eighteen healthy adults significantly decreased TxA_2_ production and showed no change in the bleeding time [120]. In a more extensive randomized, double-blind study, Eschen et al. (2004) reported that EPA (3 gm) and DHA (2.9 gm) per day to sixty healthy adults for 12 weeks significantly decreased plasma P-selectin levels, used as an index of platelet activation [121]. Consuming omega-3 fatty acids for four weeks reduced platelet integrin (GPIIa/IIIb) activation, levels of fibrinogen, and factor V, but no effect on coagulation in healthy subjects [122]. In 2008, Din and colleagues showed that 500 gm per day of mackerel consumption for four weeks reduced platelet-monocyte aggregation by ~35%, which returned to baseline after cessation of fish consumption [123]. However, Eschen et al. (2004) found no change in soluble P-selectin levels [121]. More recently, an open-label 4-week sequential study showed that EPA (1.86 gm) and DHA (1.5 gm) per day supplementation to thirty healthy adults decreased GPIIa/IIIb activation and significantly reduced the expression of P-selectin [124]. Vericel and colleagues (1999) demonstrated that a low dose of EPA (30 mg) plus DHA (150 mg) per day for 42 days resulted in a reduction in platelet aggregation but not significantly [125]. These data contrast with those published by Croset et al. (1990) [126] and Driss et al. (1984) [127]. Therein, both investigators showed that low supplementation of EPA in elderly subjects significantly decreased platelet aggregation. In the study by Croset et al. (1990) [126], healthy elderly subjects were provided with the EPA from (1984), which provided 150 mg EPA per day for one month [127]. The discrepancy in the findings between the Vericel et al. study (1999) and that of Driss and colleagues (1984) and Croset and colleagues (1990) is most likely due to the level of EPA supplementation. In 2009, Guillot and coworkers [128] demonstrated that 400 and 800 mg DHA per day for two weeks to twelve healthy elderly subjects significantly reduced platelet reactivity and TxA_2_ production. Still, this effect was reversed when 1600 mg DHA per day was consumed. DHA intake at 200 mg/day did not affect platelet reactivity or TxA_2_ production. The lack of an effect of very high intakes of DHA and EPA in healthy elderly subjects on platelet activity is consistent with findings from Larson et al. (2008), who showed no inhibition of platelet aggregation with 4 gm of omega-3 LCPUFAs per day for 4 weeks [124]. Most studies support the antiplatelet effect of marine fish or oils in healthy human subjects; although the dose used appears to be key in dictating a benefit, low and high doses do not always show a benefit.

Indeed, a meta-analysis of 15 randomized controlled trials in humans has confirmed that omega-3 LCPUFAs inhibit platelet aggregation [110]. EPA has also reduced P-selectin, oxidized LDL (ox-LDL) antibodies, and glycoprotein IIb/IIIa expression on the platelet membranes [129]. Consuming 6.6 g of omega-3 LCPUFAs reduced serum P-selectin expression, which may help to decrease platelet activity [121]. However, the increase in the n-6/n-3 ratio has shifted the balance into a proaggregatory state.

The data on the primary and secondary prevention of CVD using omega-3 LCPUFAs are controversial. The latest meta-analysis suggests that regular intake of fish oil may be a risk factor for atrial fibrillation and stroke among the general population. However, it may be beneficial in certain CVD cases [130]. The published data support the use of omega-3 LCPUFAs in reducing the risk of CVD or CVD-related death. The expert guidelines consistently recommend consuming at least 250 mg/day of omega-3 LCPUFAs or at least two servings/week of oily fish for the general population [131]. However, the beneficial role of omega-3 LCPUFAs in CVD is still not sure [132,133,134]. The clinical trials showed limited or no effect on cardiovascular health, which may result from heterogeneous populations, background omega-3 LCPUFA status, and consumption discrepancies [135].

However, it is considered appropriate for all individuals to consume omega-3 LCPUFA daily as there are some benefits associated with CVD risk reduction and no adverse effects at the recommended levels. Epidemiological and intervention studies showed that dietary intakes between 0.4 g and 1.8 g/day of omega-3 LCPUFAs are optimum for cardiovascular health. Several systematic reviews, meta-analyses, and experts examined the role of omega-3 LCPUFAs on the cardiovascular system [131,136,137,138,139,140,141,142]. Expert recommendations generally support the cardiovascular health benefit of omega-3 LCPUFAs with a recommendation for a daily intake of 500 mg as DHA and EPA or 1–2 servings of seafood per week. The US Dietary Advisory Committee recommends at least two servings of seafood per week. American Heart Association recommends one to two seafood meals per week to prevent the risk of congestive heart failure, coronary heart disease, ischemic stroke, and sudden cardiac death [138].

Meta-analyses of several randomized trials indicated that omega-3 LCPUFAs positively affect CVD outcomes for primary and secondary prevention. Marine omega-3 LCPUFAs also improve CVD risk factors, including blood pressure, plasma lipids, and inflammation; however, many physicians do not recommend omega-3 fatty acids, mainly due to controversial results in randomized trials. 

## 5. Plant Polyphenols

Plant polyphenols protect the cardiovascular system by reducing platelet hyperactivity and oxidative stress [143]. Since oxidative stress triggers inflammatory disorders, the basis for developing diseases such as immune disorders, atherosclerotic lesions, and plaque rupture, antioxidants are believed to slow the progression of many diseases, including CVD. Polyphenols can impart several health benefits, including protection from atherosclerosis, thrombosis, inflammation, and platelet hyperactivity [143,144,145,146]. The amount of polyphenols varies in plant-based foods, including fruits, chocolate, beverages, vegetables, and grains [101]. All polyphenols have the basic structure of an aromatic ring with a hydroxyl group. Polyphenols are mainly two groups, flavonoids, and nonflavonoids, despite numerous classes based on their carbon skeleton, phenol rings, and structural elements [101]. Flavonoids are further classified into flavonols, isoflavones, flavanones, anthocyanins, and flav-3-ols, whereas non-flavonoids consist of phenolic acids, hydrolyzable tannins, and stilbenes.

There is accumulating epidemiological and clinical evidence demonstrating that flavanol-rich foods can positively influence hemostasis through mechanisms that directly or indirectly affect platelet function and, in this way, ultimately decrease the risk for hypertension and stroke [147,148]. Plant-derived foods and beverages like red wine, tea, grapes, grape juice, cocoa, and chocolate are typically flavonol-rich and affect platelet aggregation.

Hamed et al. (2008) proposed that flavonoids can competitively inhibit binding to platelet-derived growth factor and TxA_2_ receptors, negating the effects of ARA—and collagen-induced platelet aggregation [149]. In addition to their antioxidant activity, flavonoids inhibit platelet lipoxygenase or reduce phospholipase C activity [150].

Most clinical studies were conducted with cocoa because cocoa products contain more flavonoids than teas and wines [151,152]. Wright et al. [153] demonstrated that functional groups of the flavonoid skeleton regulated the polyphenol inhibition of collagen-induced aggregation. The flavonoid aglycone increases the hydroxyl groups on the A and B rings, decreasing flavonoid activity. In contrast, the presence of O-methyl groups can increase activity. On the other hand, a hydroxyl group at position C3 can increase flavonoid activity [154]. In a study of 30 healthy adults, Rein et al. (2000) demonstrated that a cocoa beverage containing 18.75 g procyanidin and a total epicatechin content of 897 mg inhibited platelet aggregation and suppressed membrane GPIIa/IIIb expression and reduced the shedding of platelet microparticles. Platelet microparticles are hemostatically active, phospholipid-rich microvesicles formed by activated platelets [155]. Using the same level of flavonoids in another study of sixteen healthy young men, Pearson and coworkers (2002) showed no effect on GPIIa/IIIb activity or P-selectin expression. Heptinstall et al. (2006) evaluated the impact of different amounts of cocoa-containing flavonoids in twelve healthy volunteers [147]. Study participants consumed 80 mg, 300 mg, 600 mg, or 900 mg cocoa flavanols. Cocoa drinks containing 600 or 900 mg of cocoa flavanols significantly inhibited platelet aggregation [147]. Tomato extract, Fruitflow^®^, is also a good source of polyphenols [156], which is discussed in a later section.

Flavonols are present in red wine, tea, grapes, grape juice, cocoa, and chocolates. Hermann and colleagues (2006) showed 40 gm of dark chocolate consumption by 20 healthy adult male smokers significantly improved flow-mediated dilation and decreased platelet adhesion [152]. Hamed and coworkers also evaluated the benefits of cocoa flavanols through dark chocolate consumption [149]. Consumption of dark chocolate (100 g) for one week (~70% cocoa; 700 mg flavonoids) significantly decreased GPIIa/IIIb expression [149]. Both the studies mentioned here are consistent with the findings of Innes et al. (2003) [157]. Consuming cocoa-related products in moderate amounts inhibited platelet aggregation [158].

Several reviews documented the impact of polyphenols on platelet function. Using different agonists, polyphenols’ antithrombotic properties are determined by measuring their inhibitory effect on whole blood or platelet-rich plasma aggregation [29,32,159,160,161]. Table 1 summarizes the impact of the consumption of polyphenols on platelet aggregation ex vivo. Cocoa is a rich flavonoid source that modulates platelet activity, including catechins, proanthocyanins, anthocyanins, and flavonol glycosides [162]. Cocoa supplementation inhibited platelet aggregation when consumed acutely or chronically [163].

Olive leaf extract contains various polyphenols and flavones that inhibit platelet aggregation and ATP release 176 The olive leaf polyphenols inhibit oxidative stress-induced platelet activation via antioxidant activity [167]. Polyphenol reduces degranulation by scavenging H_2_O_2_, a substrate involved in COX-1 activation. Nevertheless, polyphenols can inhibit aggregation and secretion by inhibiting intracellular pathways in platelets.

Chlorogenic acid present in fruits inhibits collagen- and ADP-induced platelet aggregation [164]. Polyphenols dose-dependently inhibit platelet aggregation and ATP release mediated via the purinergic receptor and GPVI pathways [164]. In vitro studies showed that ellagic acid modulates platelet intracellular signaling pathways involving PLC/PKC pathways. 

Zhou et al. [165] reported that anthocyanin cyanidin-3-glucoside (C3G) significantly attenuated thrombin-induced platelet activation and ATP secretion in mice fed with high-fat diets. C3G also inhibited thrombin and collagen-induced platelet aggregation [175]. Other low bioavailable polyphenols also had antiplatelet activity [176].

Hydroxytyrosol (HT) acetate and aspirin have similar antiaggregatory activity [170]. However, HT acetate had higher antiaggregation activity than HT. A more significant antiaggregation impact of aspirin and HT acetate was observed in whole blood aggregation induced by ADP or collagen compared with aggregation in platelet-rich plasma, indicating an alternative aggregation pathway involving leucocytes, erythrocytes, platelets, and other plasma proteins. Aspirin and the two phenolic compounds increase NO production in leucocytes, thus contributing to the antiaggregation effect. NO can also affect platelet aggregation by modulating cAMP, cGMP, and TxA_2_ synthesis. Propolis inhibited ADP-induced platelet aggregation via the P2Y12 pathway [171] by decreasing the expression of the fibrinogen receptor (GPIIb/IIIa) [172].

Gallic acid inhibits ADP-induced platelet aggregation via decreasing intracellular Ca^2+^ levels, reducing phosphorylation and P-selectin secretion [173]. Olas et al. [174] demonstrated that grape seed extracts (gallic acid, falvan-3-ols, and proanthocyanins) inhibited thrombin- and thrombin receptor-activation peptide (TRAP)-induced platelet activation and platelet microparticle formation more than resveratrol [177]. Furthermore, resveratrol and its analog isorhapontigenin inhibited ADP-induced platelet aggregation [175]. Polyphenols inhibit the COX-1 and P2Y_12_ pathways and may also attenuate platelet microparticles generation in activated platelets [176]. Several polyphenols in grape seed extracts may interact with different pathways involved in platelet function. Table 1 shows the ex vivo effects of polyphenols on platelet aggregation in animal and human trials.

Trimethyl-N-oxide (TMAO) is involved in the pathology of various diseases, including cancer and CVD [178]. TMA is synthesized by the gut microbiota using dietary choline and L-carnitine as substrate; then, it is carried into the liver and converted to TMAO by flavin-containing monooxygenase 3 [178]. The conversion of choline and carnitine to TMA depends on gut microbiota diversity [178]. Therefore, gut dysbiosis increases high plasma TMAO levels and thus may ultimately result in atherosclerosis and CVD. TMAO induces platelet hyperreactivity and dyslipidemia and enhances platelet-mediated diseases such as atherosclerosis, insulin resistance, CVD, and other diseases [179,180]. TMAO enhances sub-maximal stimulus-dependent platelet activation by agonists through increased Ca^2+^ release [179]. Gut dysbiosis promotes atherosclerosis by increasing the production of TMAO [181,182,183]. Gut metagenome analysis showed a relatively lower abundance of *Roseburia* and *Eubacterium*, while TMA-producing *Collinsella* was higher in CVD patients [184]. Targeting the gut microbiota and its metabolites can effectively treat and prevent CVD by modulating TMAO levels [185,186]. Several polyphenol-rich products lower plasma TMAO levels both in animals and humans [187,188,189]. Chen et al. [187] first showed that resveratrol decreases atherosclerosis via reduced production of TMAO synthesis via remodeling gut microbiota. This observation was corroborated in rodents [188]. A study involving 20 normal-weight subjects showed a significant decrease in serum TMAO after four weeks of supplementation with 300 mg of polyphenol-rich grape pomace [189]. The polyphenol-rich extract tomato extracts lowered plasma TMAO in obese adults via modulation of gut microbiota [156]. 

## 6. Water-Soluble Extracts from Tomato and Kiwifruit: The Latest Dietary Antiplatelet Regimes

Hyperlipzidemia, hypertension, and hyperactivity of blood platelets are critical contributors to the pathogenesis of CVD [190]. Hyperactive platelets, as observed in diabetes mellitus, insulin resistance, obesity, sedentary life, and smoking, contribute to the development of CVD [191,192,193,194,195,196]. Hyperactivity of platelets contributes to the progression of atherosclerotic plaque and other diseases [108,197]. Therefore, there is a need to decrease platelet hyperactivity through safe, effective, and practical approaches to prevent the risk of CVD and other platelet-mediated diseases. Consumption of fruits and vegetables protects against CVD [198,199,200]. The phytochemicals in fruits and vegetables may protect the cardiovascular system via different mechanisms, such as favorably modulating oxidative stress, plasma lipid levels, hypertension, normalizing platelet hyperactivity, and other CVD risk factors [31,201]. For example, plant flavonoids inhibit cyclic nucleotide phosphodiesterase enzyme and platelet TxA_2_ synthesis, the main mechanisms responsible for inhibiting platelet activation.

Consequently, these phytochemicals in fruits may reduce more than one CVD risk factor, such as platelet hyperactivity [29,202]. Such nutritional antiplatelet regimes were identified in the water-soluble extract in tomatoes and kiwifruits [33,203,204]. The aqueous extract of tomatoes, also known as Fruitflow^®^, has undergone the most extensive preclinical and clinical testing to demonstrate its efficacy in inhibiting platelet hyperactivity. Recent studies showed that tamarillo, horned melon (kiwano), and raspberry extracts inhibited ADP-induced platelet aggregation [205]. 

## 7. Tomato

Epidemiological studies demonstrated that consumption of tomato and tomato products is associated with a reduced risk of CVD [206,207,208]. Tomatoes contain several compounds that might affect plasma lipids and platelet aggregation. During the last two decades, numerous studies have demonstrated that tomato’s water-soluble components inhibit blood platelet aggregation both in vitro and in vivo [30,33,204,209]. This water-soluble tomato extract (trade name Fruitflow^®^) was the first food ingredient to be given a European Commission-authorized health claim under the “emerging science” category after evaluation by EFSA in 2009 (EFSA Journal 2010). The approved claim was “Helps to maintain normal platelet aggregation, which contributes to healthy blood flow”. Fruitflow^®^ is now an established naturally derived functional food ingredient marketed globally. Multiple human studies have shown that consumption of Fruitflow^®^ causes a platelet suppression of up to 25% relative to baseline status [30].

Among all the fruits tested, the aqueous extract of tomato and kiwifruit inhibited platelet aggregation by 75–80%, whereas apple and pear had very little activity (2–5%) [31,201,203]. These tomato antiplatelet compounds had a molecular mass of less than 1000 Da and were highly water soluble and stable to boiling [33]. After removing sugars, the isolated active tomato extract accounted for 4% of the aqueous tomato extract dry matter, later named Fruitflow^®^. Sugar-free aqueous tomato (later named Fruitflow^®^ extract) showed potent inhibition of platelet aggregation in vitro [210]. Fruitflow^®^ extract had three categories of compounds: nucleosides, simple phenolic derivatives, and flavonoid derivatives.

Though the mechanisms are not yet well known, Fruitflow^®^ inhibits the GPIIb/IIIa activation step [210]. Fruitflow^®^ unaltered basal platelet cyclic AMP levels in vitro studies. In addition, Fruitflow^®^ decreased the expression of P-selectin in the platelet membranes in response to ADP in whole blood [204,210]. Fruitflow^®^ can strongly affect the size and longevity of platelet aggregates. Fruitflow^®^ was also found to affect tissue factor (TF) binding to activated platelets, at least in part due to effects on P-selectin. These results demonstrate that the actions of Fruitflow^®^ were consistent and mediated partly through polyphenols [201,211]. Effects on TF binding suggested that sugar-free aqueous tomato extract components could significantly impact some aspects of coagulation, such as thrombin generation. Fruitflow^®^ inhibits platelet granule secretion by suppressing the Src-PLCγ2-PKC-mediated granule secretory pathway. Platelet granule secretion occurs when collagen and thrombin bind to their receptors, GPVI and PARS, which causes activation of the downstream signaling pathway [212]. In this pathway, sarcoma, a tyrosine-protein kinase (Src) family member such as Lyn, Fyn, and Src, is first activated by phosphorylation, activated Syk, then phosphorylate and activated LAT, which in turn causes the phosphorylation of the adaptor protein PLCγ2 in which protein kinase C (PKC) bind leads to the activation of downstream effector, which induces platelet granule secretion. One study reported that Fruitflow^®^ has the ability to alter the generation of different interleukins such as IL-1β, IL-6, IL-10, and IL-12 and chemokines such as CCL2/MCP-1, CCL3/MIP-1α, CCL5/RANTES, CXCL8/IL-8, and CXCL10/IP-10 of blood leukocytes [212]. Fruitflow^®^ interacts with immune cells and endothelial cells [213]. It distinctly modulates the production of inflammatory modulators in a context-specific way and in extenso presumably in different body compartments.

Consequently, Fruitflow^®^ may beneficially enhance and attenuate inflammatory processes during acute and chronic inflammation. Fruitflow^®^ modulates cytokines, chemokines, and adhesion molecules that produce cell trafficking, activation, and differentiation in different immune system compartments. However, the modulatory roles of Fruitflow^®^ in immune and inflammation responses in platelets are yet to be known. Fruitflow^®^ is expected to have similar effects in platelets as those observed in different immune cells.

Fruitflow^®^ prevents the activation of integrin αIIbß3 (GPIIb/IIIa) [204]. Fruitflow^®^ also inhibited platelet aggregation and P-selectin expression in collagen-stimulated human platelets [214]. A proteomics study demonstrated that compared with the Fruitflow^®^-treated collagen-stimulated platelets, only collagen-stimulated platelets, 60 proteins, were upregulated, and 10 proteins were downregulated. Additionally, 66 phosphorylated peptides were upregulated, whereas 37 were downregulated. Proteomic studies also demonstrated that Fruitflow^®^ also strongly affected protein disulfide isomerase (PDI), an oxidoreductase that catalyzes disulfide bond formation and isomerization. In platelets, blocking PDI with inhibitory antibodies inhibits several platelet activation pathways, including aggregation, secretion, and fibrinogen binding [215,216]. Glycosides related to quercetin in Fruitflow^®^ demonstrated that they interact with PDI in this way [217,218]. The interaction of polyphenols with PDI suggested a possible mechanism by which Fruitflow^®^ ingredients inhibit different platelet activation pathways. 

Fruitflow^®^ inhibited platelet function, possibly via suppression of Akt, GSK3β, p38 MAPK, and Hsp27 phosphorylation in collagen-induced platelet aggregation. The inhibition of platelet aggregation and modification of platelet proteins in collagen-stimulated platelets by Fruitflow^®^ suggest that it can provide health benefits for people at risk of platelet hyperactivity-related thrombosis.

## 8. Human Trials and Animal Experiments Using Fruitflow^®^

Several human trials were conducted using Fruitflow^®^ to determine the ex vivo and the onset time of an acute antiplatelet effect after consumption [30,210]. An acute lowering of the platelet aggregation response to ADP and collagen was observed three hours after consuming Fruitflow^®^. The range of onset times was from one and a half hours to three hours after Fruitflow^®^ consumption. On average, these studies have shown an inhibition of the platelet response to ADP of approximately 17–25%, and platelet aggregability returned to baseline level 18 h after consumption of a single dose (150 mg) of Fruitflow^®^ [209]. A study involving 93 men and women showed that men responded more than women, and people with higher risk factors for CVD were more responsive than others [219]. Fruitflow^®^ ingredients affect many aspects of platelet function, including thrombin generation. However, clotting time showed no significant increases from baseline levels in all intervention studies [30].

A human trial demonstrated that the efficacious range for Fruitflow^®^ lies between 75 mg and 300 mg [220]. A single dose of Fruitflow^®^ was compared with 75 mg aspirin, either as a single dose or taken continuously for one week, and the platelet aggregability response was measured ex vivo [221]. The effects of a single dose of Fruitflow^®^ were similar to a 75 mg dose of aspirin in terms of antiplatelet action, effects on thromboxane synthesis, and time to form a primary hemostatic clot in a study involving 47 healthy subjects [221]. Unlike aspirin, Fruitflow^®^’s effects were not cumulative as its effects did not irreversibly disable platelet signaling pathways [221]. The antiplatelet effects of aspirin in healthy subjects are incredibly heterogeneous, with some subjects experiencing a massive increase in time to form a primary hemostatic clot while others respond poorly. The more moderate effects of Fruitflow^®^ were related to the reversibility of its antiplatelet action, rendering its use for the primary prevention of CVD, in contrast to aspirin at any dosage.

Both Fruitflow^®^ and aspirin affected 26 platelet proteins involved in platelet structure and function, coagulation, platelet secretory proteins, fibrinogen beta chain 5, Ras-related proteins, redox system proteins, and HSP70s. As described earlier, the platelet protein disulfide isomerase was affected by Fruitflow^®^, which regulates α_IIb_β_3_ integrin activation. 

Four weeks of supplementation with Fruitflow^®^ decreased platelet aggregation and granule secretion in healthy Chinese middle-aged and older individuals [222]. After a 2-week washout period, the platelet suppressive effect of Fruitflow^®^ had disappeared. After four weeks of Fruitflow^®^ treatment in the obese subgroup (n = 14), significant declines in aspirin reaction units by 8.6% and in P2Y12 reaction units (PRU) by 7.5% were observed [223]. Patients with arterial hypertension and obesity may potentially benefit from consuming Fruitflow^®^ [223].

The supplementation of Fruitflow^®^ for four weeks in strenuous exercise rats reduced platelet hyperreactivity [224]. Fruitflow^®^ treatment also reduced black tail length, blood flow pulse index, and vascular resistance index, ameliorating microcirculation perfusion in a rat thrombosis model. Fruitflow^®^ inhibited platelet aggregation induced by shear flow and alleviated the blood flow and microcirculation abnormities.

The EFSA authorized Fruitflow^®^ for daily consumption in 2009 [30]. The first Article 13 claim based on newly developed evidence or proprietary data (a particular category under Article 13(5)) was approved for Fruitflow^®^ in 2009 [30]. The approved claim was based on the eight human studies (seven proprietaries) and seven non-human studies (three proprietaries) conducted with Fruitflow^®^.

Fruitflow^®^ differs fundamentally from aspirin or other antiplatelet drugs in that its effects are reversible. This very significant property of Fruitflow^®^ makes it suitable for use by the general population as a dietary functional ingredient, while antiplatelet drugs cannot be used.

## 9. Kiwifruit and Its Antiplatelet Factors

Kiwifruit contains substantial quantities of several bioactive polyphenols [220]. Kiwifruits contain significant amounts of vitamin C, E, folic acid, and various phytochemicals, such as anthocyanidins and flavonols. Some of these polyphenols in this fruit are known for their antiplatelet activity [4,220]. The familiar green kiwifruit, *Actinidia deliciosa*, has been investigated for bioactivity, especially its effects on the risk of CVD [31,225,226,227]. As in tomato extract, the aqueous extract of kiwifruits has maximum antiplatelet factors, which was unrelated to the fruits’ antioxidant potential [201]. Daily consumption of two or three kiwifruits reduced ex vivo platelet aggregation response, blood pressure, and plasma lipids [31,225,226,227]. The diverse activities of the aqueous extract of kiwifruit, such as the lowering of antiplatelet, plasma triglycerides and the converting of enzyme (ACE) activity by anti-angiotensin, have been shown [31,228]. The aqueous kiwifruit extract (KFE) inhibited ADP- and collagen-induced platelet aggregation.

In contrast, KFE was not that effective against ARA-induced platelet aggregation. KFE-mediated inhibition of platelet aggregation may not involve the TxA_2_ pathway. The antiplatelet compounds in kiwifruit are water soluble and heat stable, and their molecular mass is less than 1000 Da [31]. KFE had glucose (8.9 ± 0.4 mg/mL), fructose (9.9 ± 0.5 mg/mL), and sucrose (2.3 ± 0.2 mg/mL) [203]. The inhibition of platelet aggregation by sugar-free KFE was concomitantly associated with the inhibition of TxB_2_ synthesis and inhibited PF4 release in a dose-dependent manner. KFE at 1.68 mg/mL inhibited ADP- and collagen-induced TxB_2_ synthesis by 91% and 81% [229]. 

## 10. Human Trials 

Several human intervention trials were conducted to investigate the ex vivo effects of whole kiwifruits and KFE on platelet aggregation, ACE, and blood pressure [31,230,231,232]. In one human trial involving 30 healthy volunteers aged 20–51 years [31], consuming two or three kiwifruits daily for 28 days reduced platelet aggregation ex vivo. Consuming two kiwifruit daily inhibited platelet aggregation induced by ADP significantly (18% in case of 4 μM ADP and 15% in case of 8 μM ADP) compared with those at day 0 (*p* < 0.05). A similar reduction in platelet aggregation was observed in response to collagen. Plasma triglyceride levels were significantly lowered on day 28. The total plasma cholesterol, LDL, and HDL levels were unchanged from days 0 to 28 in both groups [31].

Another human trial showed that consuming one kiwifruit daily for four weeks reduced whole-blood platelet aggregation in healthy volunteers [230]. The effects of consumption of three kiwifruit for eight weeks on blood pressure, plasma lipids, and whole-blood aggregation were investigated in a randomized, controlled trial in male smokers (aged 44–74 years) [232]. In the kiwifruit group, reductions of 10 mm Hg in systolic blood pressure and 9 mm Hg in diastolic blood pressure were observed [232]. Additionally, a 15% reduction in whole-blood aggregation and an 11% reduction in ACE activity were observed in the kiwifruit group [232]. Although only blood pressure was decreased in hypertensives, no effects on other parameters were observed in the antioxidant-rich diet group. These diverse activities of kiwifruits, such as antiplatelet and anti-ACE, were also demonstrated in differently processed aqueous extracts of kiwifruits [31,228]. These results bear on not only green kiwifruits: consuming one gold kiwifruit daily for four weeks also reduced whole-blood platelet aggregation and plasma triglycerides in healthy volunteers [230]. Consuming 2 mg of KFE in 10 g margarine inhibited ex vivo platelet aggregation by 12.7% two hours after consumption by healthy volunteers (n = 9) [203].

## 11. Conclusions

Many studies have explored the relationship between dietary components, platelet function, and CVD risk factors. Several antiplatelet regimes, such as polyphenols, omega-3 fatty acids, and fruits and vegetables, have been developed. The overall data are positive for nutritional antiplatelets (such as fish and fish oil, polyphenols, Fruitflow, and KFE) to prevent CVD. However, platelet hyperactivity is also associated with many non-hemostatic diseases. Therefore, the impacts of these antiplatelet dietary factors on these diseases must be further investigated. The next question is, which of these nutritional antiplatelets is superior in its efficacy as an antiplatelet ingredient? At present, Fruitflow^®^ is the most studied potent antiplatelet functional food. Fruitflow^®^, developed from tomato-containing bioavailable cardioprotective compounds, can benefit people vulnerable to developing CVD.

Beyond efficacy, one must also consider the practical aspects of consuming such dietary antiplatelets daily. For example, dosing, product form, commercial viability, and cost should also be considered. Finally, reliable markers of platelet activity and blood flow and the techniques thereof need to be further studied. Platelets become hyperactive in obesity, diabetes, a sedentary lifestyle, or hypertension, and in people who smoke. Platelet hyperreactivity is not merely an inevitable adverse effect of conditions but also a substantial contributing factor that exacerbates most human pathologies [29,194,233]. Recent studies indicate that platelet hyperactivity is not just a biomarker of adverse effects but a condition that requires management. Modulating platelet reactivity towards collagen, ADP, and plasma triglyceride levels by functional foods could be of potential prophylactic and therapeutic benefit in preventing and halting pathologic processes.

An array of extensive basic, mechanistic, compositional, and several human trials testify to the cardio-protective benefits of Fruitflow^®^ [31,201,211,234]. In addition to Fruitflow^®^, Kiwifruits have great potential for maintaining normal platelet activity.

It is now recognized that 20–30% of persons experience so-called aspirin-resistance syndrome, in which the expected antiplatelet effects are not observed [235]. This finding indicates an advantage of functional foods’ broad antiplatelet activity profile over single-target drugs such as aspirin. Dietary antiplatelet functional foods help provide suitably gentle and safe yet efficacious therapies to improve public health in response to various health challenges involving platelet hyperactivity. Evidence from interventional trials have shown that consuming certain functional foods may improve cardiovascular health through their antiplatelet properties. However, further work is required on the beneficial effects of other platelet-mediated diseases such as cancer metastasis, immune disorders, hypertension, diabetes, inflammatory diseases, rheumatoid arthritis, myeloproliferative disease, and Alzheimer’s disease. Well-controlled studies on food sources and their impacts on these diseases will help improve our understanding of potential antiplatelet effects on health and disease.

## Figures and Tables

**Table 1:** Ex vivo effects of polyphenols on platelet aggregation in animals and human trials.

Source	Models	Experimental Conditions	Conclusions
Chlorogenic acid	Mice (n = 18)	Inhibition of in vivo thrombus formation	Inhibited thrombus formation [164].
Anthocyanin cyanidin-3-glucoside from purified black rice	Mice (n = 60)	Mice randomly assigned to 3 groups (n = 20): control group, high-fat diet, or a high-fat diet + Anthocyanin cyanidin-3-glucoside	Decreased platelet activation, and inhibited platelet ATP release [165].
Red wine polyphenols (Provinols™)	Rats (n = 149)	Rats were randomly grouped as treated with or without aldosterone–salt, with or without Provinols (20 mg/kg/day) or spironolactone (30 mg/kg/day) for 4 weeks	Provinols decreased circulating levels microparticles [166].
Cocoa	Healthy males (n = 16)	Double-blind, crossover study. Placebo-controlled. Eight subjects in two groups (trained and untrained) randomly received placebo or cocoa polyphenol (236 mg/day) for a week and then afterwards were subjected to one hour of exercise	No change in collagen induced platelet aggregation post-exercise. ATP release higher post-exercise in both groups. Cocoa supplementation did not normalize platelet activity after exercise [167].
Chicory coffee	Healthy subjects (n = 27)	Chicory coffee (300 mL) consumed every day for 1 week	Effect on platelet aggregation is variable depending on the agonists used [168].
High polyphenol beverage	Healthy Athletes (n = 103)	Group 1 received a polyphenol-rich beverage, Group 2 a placebo in a randomized, double-blind study. Samples were collected three weeks before, one day before, immediately, and 24 h and 72 h after a marathon run	Control group demonstrated a 2.2-fold increase in platelet aggregation after marathon completion. But there was no increase in platelet aggregation in polyphenol-rich beverage group [169].
Polyphenol-rich grape wine	Untreated, mildly hypertensive subjects (n = 60)	Grape juice extract; grape and wine extract each for 4 weeks including a 2-week run-in period in a double-blind placebo-controlled crossover study	There was no effect on ADP, collagen, or epinephrine induced platelet aggregation [170].
Polyphenol-rich grape seed extract	Untreated subjects with pre- and stage 1 hypertension (n = 35)	Double-blind, placebo-controlled, randomized, parallel-group intervention with 300 mg/day grape seed extract capsule. Eight-week duration study	Platelet aggregation was not affected [171].
Flavanol-rich chocolate	Patients with congestive heart failure (n = 20)	A total of 2 h after consumption of a chocolate bar and 4 weeks of consumption (two chocolate bars/day) in a double-blind, randomized placebo-controlled trial	Platelet adhesion significantly decreased 2 h after flavanol-rich chocolate ingestion. But no effect after 2- and 4-week supplementation [172].
Chokeberry (*Aronia mitschurinii*) products	Patients with untreated mild hypertension (n = 38)	Cold-pressed 100% chokeberry juice (300 mL/day) and oven-dried chokeberry powder (3 g/day), or placebo for 8 weeks without washout in a single-blinded crossover trial for 16 weeks	No change in platelet aggregation response [173].
Oats	Type 2 diabetes subjects (n = 22)	Randomized crossover involving 8-week intervention with either oat-enriched diet or a standard dietary advised diet. Pre-intervention habitual intakes were used to compare responses	Decreased in tissue factor-activated platelets (CD142) after oat-rich diet than habitual or standard advised diet. Decreased in tissue factor-positive platelet microparticles and fibrinogen-positive platelet microparticles with oat-enriched diet intervention [174].
Anthocyanin-rich beverage	Sedentary subjects (n = 21)	Queen garnet plum juice (200 mL/day) were consumed for 28 days in a double-blind placebo-controlled study	Reduced ADP-, collagen-, and ARA-induced platelet aggregation. Reduced P-selectin expression.

## Abbreviations

CVD: cardiovascular disease; EPA, eicosapentaenoic acid, 20:5n-3; ARA, arachidonic acid, 20:4n-6; DHA, docosahexaenoic, 22:6n-3; EFSA, European Food Safety Authority; PDI, protein disulphide isomerase; TF; tissue factor, PRP, platelet-rich plasma; TxA_2,_ thromboxane A_2_.
